# Sleep as a Mediator in the Pathway Linking Environmental Factors to Hypertension: A Review of the Literature

**DOI:** 10.1155/2015/926414

**Published:** 2015-03-02

**Authors:** Oluwaseun A. Akinseye, Stephen K. Williams, Azizi Seixas, Seithikurippu R. Pandi-Perumal, Julian Vallon, Ferdinand Zizi, Girardin Jean-Louis

**Affiliations:** ^1^Department of Medicine, Icahn School of Medicine at Mount Sinai, Queens Hospital Center, 82-68 164th Street, Jamaica, NY 11432, USA; ^2^Center for Healthful Behavior Change, Department of Population Health, NYU School of Medicine, 227 East 30th Street, New York, NY 10016, USA

## Abstract

Environmental factors, such as noise exposure and air pollution, are associated with hypertension. These environmental factors also affect sleep quality. Given the growing evidence linking sleep quality with hypertension, the purpose of this review is to investigate the role of sleep as a key mediator in the association between hypertension and environmental factors. Through this narrative review of the extant literature, we highlight that poor sleep quality mediates the relationship between environmental factors and hypertension. The conceptual model proposed in this review offers opportunities to address healthcare disparities in hypertension among African Americans by highlighting the disparate impact that the predictors (environmental factors) and mediator (sleep) have on the African-American community. Understanding the impact of these factors is crucial since the main outcome variable (hypertension) severely burdens the African-American community.

## 1. Introduction

Chronic noise exposure and air pollution have become a significant public health issue given its ubiquity in urban environments [1, 2]. For the first time in history, more than 50% of the world's population lives in an urban environment, and it is estimated that it will likely rise to 70% by 2050 [1]. Urban environments teeming with subways, airplanes, road traffic, and emergency vehicle sirens are epicenters of chronic noise and pollutant exposure [3]. The health implications of chronic exposure to noise and pollutants are becoming more evident based on increased levels of related chronic disease, deaths, and impaired quality of life issues in urban populations. Noise complaints in New York City (NYC) were the primary insult to quality of life in the late 90s [4]. It is estimated that air pollution is responsible for 6% of annual deaths in NYC [5].

Although the association between hypertension and environmental factors, such as ambient noise exposure [6] and air pollutants [7, 8], is well documented primarily in laboratory-based studies [9–12], field-based studies accounting for chronic exposures will likely provide more clinically and ecologically relevant hypertension risk factors. Hypertension has a multifactorial etiology [13] and sleep quality has been identified as a factor associated with hypertension [14–17]. Hypertension and sleep disorders are common in the USA. It is estimated that 60–70 million Americans suffer from hypertension [18] and a similar number of Americans also suffer from chronic inadequate sleep, hindering daily functioning and adversely affecting health and longevity [19].

**Figure 1:**
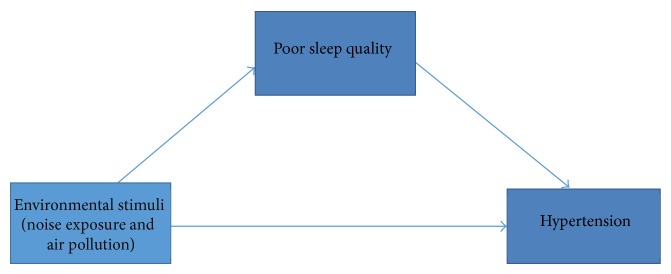
Conceptual model proposing sleep quality as a mediator in the pathway linking environmental stimuli to hypertension.

The theory that sleep could be a mediator in the role between environmental factors and cardiovascular health outcomes has been proposed in the past [20]. As per the Baron and Kenny model [21], to satisfy the conceptual model for mediating a predictor-outcome relationship, the following relationships must be present: (1) The proposed environmental factors (predictor variables) are significantly associated with hypertension (outcome variable); (2) the environmental factors must be significantly associated with sleep (mediating variable); and (3) sleep must be associated with hypertension (Figure 1). In a previous study, noise sensitivity and health complaints were noted to be associated and sleep was found to be a mediating variable [22]. Additionally, noisy neighborhoods were associated with lower self-rated physical health and the association was also mediated by sleep [23]. Conversely, in a study evaluating the adverse impact of night-time aircraft noise on endothelial function, sleep quality was not noted to be a mediating factor [24].

The purpose of this review is to summarize: (1) the relationship of hypertension to noise and air pollutants; (2) the relationship between sleep and the aforementioned environmental factors; and (3) the relationship between hypertension and sleep. We propose that relationships between these environmental factors and hypertension among Blacks would be especially relevant clinically because of the greater exposure to the aforementioned environmental factors as well as the greater prevalence of poor sleep quality in that population.

## 2. Sleep and Hypertension

Sleep has become of great interest in the field of hypertension because of the potential interventions that could be administered with resultant improvement in hypertension and its related adverse outcomes. The CARDIA Sleep Study, a longitudinal study examining the association between hypertension and sleep quality, showed an increase in the odds of hypertension among individuals with shorter sleep duration [25]. Data from a cross-sectional analysis of the 2009 National Health Interview Survey (NHIS) showed a higher likelihood of hypertension among individuals who reported daily sleep durations of 6 hours or less [26]. This is consistent with findings from the National Health and Nutrition Examination Survey (NHANES) I Epidemiologic Follow-Up Study that revealed a hazard ratio of 1.60 (95% CI: 1.19, 2.14) for developing hypertension over 8 to 10 years among individuals reporting sleep duration of 5 hours or less compared with those sleeping 7 to 8 hours [15].

## 3. Environmental Factors and Hypertension

Environmental factors are included under the social determinants of health and are emphasized in the Healthy People 2020 initiative [27]. The environmental factors that this review will emphasize are limited to chronic ambient noise exposure (nonoccupational) and chronic ambient air pollution (Table 1). The air pollutants that will be discussed are predominantly PM_2.5_, which is defined as fine particulate matter <2.5 *μ*m in diameter.

## 4. Noise and Hypertension

“Noise is generally defined as unwanted sound or set of sounds.” [28] Noise disturbance is one of the most frequent complaints among urban-dwelling populations [28] and can be due to amplification of the noise levels by reflections off rigid urban area structures [29]. These areas are also more likely to have higher volume of vehicular traffic, train tracks, and airports [30]. Chronic exposure to subway traffic noise in New York City has been documented to have potential for significant adverse health effects with decibel levels exceeding 100 dB(A) [30, 31]. Decibel levels > 70 dB(A) are thought to be associated with harmful cardiovascular health effects [32].

The epidemiologic evidence associating noise with higher risk of developing hypertension continues to grow [33–35]. The Hypertension and Exposure to Noise near Airport (HYENA) study, comprising almost 5,000 participants living close to several European airports, showed a significant exposure-response relationship between night-time aircraft, average daily road traffic noise exposure, and risk of hypertension [36]. Evidence shows a 1.14 (95% CI: 1.01, 1.29) increase in the odds of developing hypertension with each 10 dB increase in exposure to night-time aircraft noise, and similar exposure-response relationships were seen for road traffic noise in the highest exposure category of >65 dB with a significant odds ratio (OR) of 1.54 (95% CI: 0.99, 2.40) [36]. Furthermore, an increase of 0.26 mmHg in systolic blood pressure (BP) per 10 dB increase in road traffic noise levels and a 8% higher risk of hypertension with exposure to railway noise > 60 dB were reported in another study [37]. A smaller study found that there were a 1.06 to 1.80 increased odds of hypertension for every 5 dB increase in noise exposure and the association was stronger among those who had lived at the same address for >10 years [38], suggesting additional impact among those with more chronic exposure. This finding was supported by another cross-sectional study that showed a stronger estimate of noise effect on the risk of hypertension in individuals with chronic noise exposure [39]. More significant estimates of the noise effect were found in subjects with long residence time (OR: 1.20, 95% CI: 1.05–1.37) and with exposure of the living room during daytime (OR 1.24, 95% CI: 1.08–1.41) compared with the exposure of the bedroom during night-time [39].

In contrast, the ROADSIDE study found no statistically significant association between road traffic noise and traffic load and self-reported hypertension [40]. This finding was reproduced by Dratva et al. [41], who showed no significant association between traffic noise and hypertension except in diabetics. Analysis of the HYENA study also supported this differential effect of the source of noise on hypertension [42]. Other studies found the association between noise and hypertension to be gender [43, 44] and age [45] dependent.

## 5. Air Pollution and Hypertension

The urbanization of most American cities is likely to worsen the problem of air pollution. In addition to soot, smog, smoke, dust, ozone, sulfur, carbon monoxide, nitrogen dioxide, and lead which make up the particulates, suspended particulates in the air come from increased combustion of motor vehicle fuel in the urbanized environment, posing a serious health threat among urban dwellers. Recommended limits for PM_2.5_ are 35 *μ*g/m^3^ daily and 15.0 *μ*g/m^3^ annually [46]. In 2000, the annual average in downtown NYC was 17.5 *μ*g/m^3^ [47]. Both indoor and outdoor pollution have been associated with adverse effects on human health [48–54]. Even though asthma is typically the disease associated with air pollution, accumulating evidence suggests that hypertension is associated with air pollution [55–58].

Data from a meta-analysis suggested that blood pressure (BP) was positively related to PM_2.5_ exposure, resulting in an increase of 1.393 mmHg and 0.895 mmHg per 10 *μ*g/m^3^ increase in PM_2.5_ exposure for systolic BP and diastolic BP, respectively [41]. Further supporting a causal mechanism was the finding that, with long-term exposure, there was a stronger association with BP increase [2]. This is consistent with findings in another study that found a 2.8 mmHg, 2.7 mmHg, and 2.7 mmHg increase in resting systolic, diastolic, and mean arterial BP, respectively, following exposure to mean PM_2.5_ level of 10.5 *μ*g/m^3^ [59]. Dvonch et al. [60] also noted similar associations between PM_2.5_ and BP, and larger effects were observed when urban location was controlled for in the analysis. An interquartile range (IQR) increase in PM_2.5_ (2.4 *μ*g/m^3^) was found to be associated with estimated increases in mean systolic and diastolic BP of 1.4 mmHg and 0.9 mmHg, respectively [61], and stronger effect sizes from chronic exposure to ambient air pollution [62] and other air pollutants [63] have been reported. There are conflicting results with the finding that long-term exposure to traffic air pollution is inversely associated with systolic and diastolic BP and the prevalence of self-reported hypertension [64]; however it should be noted that there were differences in the method that traffic air pollution was assessed in this study. The fact that nitric oxide (NO), but not PM_2.5_, was assessed makes direct comparison with the majority of the traffic air pollution studies difficult. It is also worthy of note that PM_2.5_ retained the strongest association with blood pressure (both systolic and diastolic) among four air pollutants (ozone (O(3)), nitrogen dioxide (NO(2)), sulfur dioxide, and carbon monoxide) in a recent study [65].

## 6. Environmental Factors and Sleep Quality

Urban communities are more likely than rural communities to have certain neighborhood characteristics (i.e., noise and air pollution) that affect sleep quality. The odds of having short sleep are highest for those who live in central city environments with over 1 million people compared with residents of more rural, nonmetropolitan environments even after adjusting for socioeconomic and health characteristics [66].

## 7. Noise and Sleep Quality

Noise is independently associated with reduced sleep quality [67, 68]. Data from a cross-sectional study showed that residing in noisy area is associated with difficulties falling asleep, falling back to sleep, waking up at night, and having poor sleep quality [69]. In a related study, chronic exposure to loud noise over a year was strongly associated with poor sleep efficiency resulting in a decrease in total rapid eye movement time, none rapid eye movement time, slow-wave sleep time, sleep onset latency, and total sleep time [70]. A Norwegian study also developed a model that presented sleep disturbance as a mediator of the noise and poor health relationship. It is interesting that a phenomenon of “noise annoyance” was found to be a mediator factor suggesting that a vulnerable subset of the population was more likely to suffer from the adverse health effects of noise [20]. Different populations have been noted to rate different sources of noise population as being noxious [68, 71]. Environmental noise elevates arousal levels and fragments sleep resulting in a redistribution of time spent in the different sleep stages [68], typically increasing wake and stage 1 sleep and decreasing slow-wave and REM sleep [72, 73]. The method by which sleep quality is assessed must be considered in examining the research literature since it has been reported that subjective assessment of sleep quality after exposure to noise does not correlate well with objective findings [68, 74].

## 8. Air Pollution and Sleep Quality

Decreases in sleep efficiency have been associated with increases in short-term variation of particulate air matter less than 10 *μ*m in aerodynamic diameter [51]. An analysis of a cross-sectional study showed a statistically significant association between higher PM_10_ exposure and disorders of initiation and maintenance of sleep assessed by the Sleep Disturbance Scale for children questionnaire [75]. Exposure to black carbon (a marker of traffic-related air pollution) in urban Boston was associated with short sleep duration in men and in the lower socioeconomic (SES) population. However, the complexities in such analysis are highlighted by associations with longer sleep duration in Blacks, an association not found in Whites or Hispanics [76].

## 9. Discussion

Even though there is accumulating evidence on the association between hypertension and environmental factors such as air and noise pollution, there remains a significant gap in elucidating the mechanism of this association. In the USA, there is a paucity of published research that has examined the association between hypertension and environmental factors with most of the cited research originating from the Scandinavian countries. This finding is somewhat surprising given the urbanization of our nation's communities. Nonetheless, these environmental factors, in particular air pollution, have become a critical issue for the American Heart Association (AHA) [54]. Given the evidence that environment plays a significant role in determining blood pressure levels and hypertension prevalence, it is imperative that the United States derived data be gathered in the interest of the nation's public health [77, 78].

The proposed model presented in this review is simple by virtue of the paucity of data on the relationships. Field studies, as opposed to laboratory studies, are indicated to correlate long-term exposure to environmental factors to hypertension and sleep. There is evidence that sleep responses to noise in the field are different to laboratory exposure [79] likely because of physiological factors such as habituation [80] and behavioral factors such as subject self-selection for living in particular locations [81]. Idiosyncratic domestic stimuli, such as bed partner movement and children going to bathrooms, have been shown to be more influential on sleep patterns than road traffic or aircraft noise [82, 83]. Future models will need to clarify the relative contributions of genetics to the environment-hypertension association.

The premise of this paper is that there is a glaring gap in extant literature investigating the mediating effect of sleep on the noise-BP association, given the well-established associations of noise-BP and noise-sleep. The HYENA study demonstrated a relationship between exposure to aircraft noise and hypertension with a stronger relationship noted with night-time exposure to noise as opposed to daytime [36]. The implication of these findings is that night-time noise disturbance may be more significant by virtue of sleep disturbance. In a separate study of exposure to aircraft noise, while greater perception of noise disturbance was associated with poorer subjective sleep quality, higher objective measures of noise were associated with higher BP levels [84]. There is even more of a paucity of clinical research when exploring the air pollution-BP and the air pollution-sleep association. An intriguing area of exploration is the common denominator of electroencephalography (EEG) arousals in both the pathogenesis of BP physiology and sleep architecture. It is well established that EEG findings are instrumental in describing sleep architecture [85]. EEG changes have been observed when individuals are exposed to air pollutants [86], and similarly EEG findings have been shown to precede changes in BP homeostasis [87]. These findings suggest a gap in our understanding of the associations between environmental factors and BP, warranting further investigations.

In the United States, there is the opportunity to link data sets such as NHANES with validated spatiotemporal models or data such as the NYC community air survey (NYCCAS) to investigate cross-sectional associations of environmental factors and hypertension. Such endeavors are in the infancy of being funded by the National Institute of Health (NIH) [88]. This recently funded study uses a cohort from Black Women's Health Study to determine the incidence of hypertension and diabetes associated with PM_2.5_ while controlling for noise confounded using innovative modelling.

Finally, the proposed model has the potential to address ethnic/racial disparities in the field of hypertension. Innovative research methods from Southern California have illustrated the excess exposure to road traffic and its pollutants that minority populations experience [89, 90]. The National Institute of Environmental Health Sciences is a branch of the NIH that has at its core mandate a stated mission to address environmental health disparities. There is a higher prevalence of hypertension in the African American population and African Americans disproportionately suffer from poor sleep quality, relative to other racial/ethnic groups [66, 91–94]. They also dwell predominantly in urban populations and are subjected to a unique array of intense and prolonged environmental stimuli. There should be opportunities for further funding from the NIH to address these relevant issues.

## 10. Conclusions

In summary, sleep as a mediator in the pathway of the relationship between environmental factors and hypertension is an intriguing model with promising avenues for therapeutic intervention. The factors comprising the conceptual model are especially common and small improvements in the proposed predictor and mediator variables could lead to significant improvements in the management of hypertension. In addition, the epidemiology of the discussed variables is such that they promise to address racial/ethnic disparities in hypertension.

## Figures and Tables

**Table 1a:** (a) Environmental noise and hypertension

First author, year (design)	Sample size (*N*)	Country	Exposure assessment method	Hypertension assessment	Main finding	Effect size: ES *r* (95% CI)
Jarup, 2008 [36] (cross-sectional)	4861	United Kingdom, Germany, Netherlands, Sweden, Italy, Greece	Country specific noise exposure models: both aircraft and road traffic noise	Home BP readings with hypertension defined as ≥140/90 mmHg OR self-reported physician diagnosis of hypertension OR use of antihypertensive medication	Significant exposure-response relationship between night-time aircraft noise, average daily road traffic noise exposure, and risk of hypertension	Night-time aircraft noise: OR 1.14 (95% CI: 1.01–1.29) of hypertension with 10 dB(A) increase in exposure; average road traffic noise: OR 1.097 (95% CI: 1.00–1.20) of hypertension with 10 dB(A) increase in exposure

Bluhm, 2007 [38] (cross-sectional)	667	Sweden	Nordic prediction model for road traffic noise	Self-report on survey	Significant relationship between exposure to residential road traffic noise and hypertension; association stronger among women and among those who have lived at the address for >10 years; exposure-response relationship suggested	The odds ratio for hypertension was 1.38 for every 5 dB(A) increase in noise exposure

Sørensen, 2011 [37] (cross-sectional)	44,083	Denmark	Nordic prediction model for road traffic noise and railway noise	Incident hypertension over 5 years identified by questionnaire; baseline association between measured blood pressure and residential exposure to road traffic noise	Exposure-response relationship between road traffic noise and systolic blood pressure levels; effect size statistically significant only in males; no association between road traffic noise levels and diastolic BP; there was a borderline statistically significant relationship between railway noise and incident hypertension cases	Cross section: increase of 0.59 mmHg in systolic BP (95% CI: 0.13–1.05) per 10 dB(A) increase in road traffic noise levels; prospective: 8% higher risk of hypertension with exposure to railway noise above 60 dB(A) (95% CI: −2%–19%)

Babisch, 2014 [39] (cross-sectional)	1770	Berlin, Germany	City noise map for road traffic noise	Self-reported physician diagnosis, use of antihypertensive medications, measured blood pressure ≥140/90	Stronger significant estimates of the noise effect were found in subjects with long residence time and with respect to the exposure of the living room during daytime, no association with respect to exposure of the bedroom during night-time	OR for developing hypertension while living at the residence was 1.11 interval (95% CI: 1.00–1.23) per noise level increment of 10 dB(A); effect size stronger with resident time >10 years (OR: 1.20; 95% CI: 1.05–1.37); OR for development of hypertension was 1.24 (95% CI: 1.08–1.41) in those living with exposure of the living room during daytime

Eriksson, 2012 [40] (cross-sectional)	25,851	Sweden	Traffic load (millions of vehicle kilometers per year) within 500 m around residential address; subpopulation had additional assessment using Nordic prediction method	Self-reported physician diagnosis of hypertension	No significant association noted between traffic load and hypertension	OR for diagnosis of HTN with exposure to ≥65 dB(A) with <50 dB(A) as reference was 0.96 (95% CI 0.59–1.59)

de Kluizenaar, 2007 [45] (cross-sectional)	2 samples Sample 1: 40,856 Sample 2: 8592	2 samples (1) City of Groningen sample (2) Sample from observational study: Prevention of Renal and Vascular End-Stage Disease (PREVEND)	Road traffic noise exposure of the subjects was calculated at the most exposed facade of the dwelling with standard method	Groningen subjects were defined as having hypertension when they reported using medication for elevated blood pressure	Adjusted ORs summarizing noise exposure and hypertension were not significant; significant findings in subjects who were between 45 and 55 years old; associations seemed to be stronger at higher noise levels	In the Groningen sample, adjusted OR for having hypertension in subjects between 45 and 55 years old was 1.19 (95% CI: 1.02–1.40) per 10 dB increase in noise level (Lden) PREVEND cohort OR 1.39 (1.08–1.77)

Haralabidis, 2011 [42] (cross-sectional)	149	4 European airports with night-time flights permitted	Long-term noise exposure as per HYENA study protocol (aircraft and road traffic)	Ambulatory blood pressure measurement for 24 hr	Only road traffic noise, and not aircraft noise, was associated with decreased BP dipping	Pooled estimates: with a 5 dB(A) increase in measured road traffic noise, there is 0.8% less dipping (95% CI: −1.55 % to −0.05%)

Dratva, 2012 [41] (cross-sectional)	6,450	Switzerland	SONBASE national data repository on railway and traffic noise linked to residential addresses	Measured at rest by study staff	Positive association of railway noise with SBP and DBP; effect size stronger among subjects with reported physician-diagnosed hypertension, DM, or CVD; traffic noise was not impressive except for people with DM	For a 10 dB(A) increase in railway noise during the night 0.84 mmHg increase in SBP (95% CI: 0.22–1.46) and during the day 0.60 mmHg increase (0.07–1.13)

Belojević, 2008 [43] (cross-sectional)	2,503	Belgrade	Grouped into noisy areas (equivalent noise level [Leq] > 45 dB(A)) and quiet areas (Leq ≤ 45 dB(A))	Use of antihypertensive medication OR measured BP with values ≥140/90 mmHg defined as hypertension	Night-time urban road traffic noise might be related to occurrence of hypertension; no significant findings in women	In men, OR for hypertension was 1.58 (95% CI: 1.03–2.42).

Barregard, 2009 [44] (cross-sectional)	1386	Sweden	GIS and validated model to assess road and railway traffic noise	Self-report of physician diagnosis or taking antihypertensive medications	Association between road traffic and hypertension noted along with an exposure-response relationship; there were no clear associations in women or for railway noise	OR for hypertension was 1.9 (95% CI: 1.1 to 3.5) in the highest noise category for road traffic noise; OR for hypertension was higher in men—3.8 (95% CI: 1.6 to 9.0)

Eriksson, 2007 [35] (prospective)	2,754 men	Stockholm airport	Geographical information systems techniques	Incidence cases of hypertension defined by self-report of physician diagnosis or use of medications or BP measured at ≥140/90 mmHg	Long-term aircraft noise exposure increases risk for hypertension	For subjects exposed to energy-averaged levels above 50 dB(A) the adjusted relative risk for hypertension was 1.19 (95% CI: 1.03–1.37); maximum aircraft noise levels presented similar results, with a relative risk of 1.20 (1.03–1.40) for those exposed above 70 dB(A)

**Table 1b:** (b) Ambient air pollution and hypertension

First author, year (design)	Sample size	Country	Air pollution assessment method	Hypertension assessment	Main finding	Effect size: ES *r* (95% CI)
Fuks, 2011 [61] (cross-sectional)	4,291	Urban West Germany	PM_2.5_ using validated dispersion model system	Measured systolic BP ≥140 mmHg or diastolic BP ≥90 mmHg or current use of antihypertensive therapy	Long-term exposure to PM_2.5_ is associated with increased blood pressure; more impressive findings with traffic noise proximity	An IQR increase in PM_2.5_ (2.4 *μ*g/m^3^) was associated with estimated increases in mean systolic and diastolic BP of 1.4 mmHg [CI 95%: 0.5–2.3] and 0.9 mmHg (CI 95% CI: 0.4–1.4), respectively

Auchincloss, 2008 [62] (cross-sectional)	5,112	North America: Multiethnic Study of Atherosclerosis (MESA)	PM_2.5_ using 24-hour integrated samplers with 5 retrospective exposure phases recorded	Resting seated BP	Stronger effect sizes from longer exposures (1-2 months) of ambient PM_2.5_ exposure compared with shorter (≤1 week) exposures; systolic blood pressure was only significantly affected (diastolic was not); effects stronger in the presence of higher traffic exposure	10 *μ*g/m^3^ increase in PM_2.5_ prior 30-day mean was associated with 2.8 mmHg SBP (95% CI: 1.38 to 4.22) **Note that this is a relatively short exposure study**

Chuang, 2011 [65] (cross-sectional)	1,023	Taiwan	1-year averaged criteria air pollutants measured by local monitoring stations (PM_2.5_, PM_10_, nitrogen dioxide (NO(2)), and ozone (O3))	Measured blood pressure	PM_2.5_ retained the strongest association with blood pressure (both systolic and diastolic) among the four air pollutants	For an IQR increase in PM_2.5_ (20.42 *μ*g/m^3^), there were 32.4 mmHg (95% CI: 22.4–42.5) and 29.3 mmHg (95% CI: 19.2–39.3) increases in systolic and diastolic blood pressure, respectively, (controlling for ozone), and 31.1 mmHg (95% CI: 21.1 to 41.2) and 30.0 mmHg (95% CI 18.0 to 41.9) increases in systolic and diastolic blood pressure, respectively, (controlling for NO(2))

Dong, 2013 [58] (cross-sectional)	24,845	China	Local monitoring stations: three-year average concentration PM_10_, sulfur dioxide (SO2), nitrogen dioxides (NO2), and ozone (O3)	Measured blood pressure	Note that these are findings for more coarse particles, comparing apples and oranges; it addresses exposures to a mixture including not only PM_2.5_	Odds ratio for hypertension increased by 1.12 (CI 95%: 1.08–1.16) per 19 *μ*g/m^3^ increase in PM_10_; the estimated increases in mean systolic and diastolic blood pressure were 0.87 mmHg (95% CI, 0.48–1.27) and 0.32 mmHg (95% CI, 0.08–0.56) per 19 *μ*g/m^3^ increase in PM_10_

Coogan, 2012 [56] (prospective)	4,204	USA	Participants' residential addresses with land use regression models (nitrogen oxides) and interpolation from monitoring station measurements (PM_2.5_)	Incident case of hypertension as self-report of physician-diagnosed hypertension during follow-up and concurrent use of antihypertensive medications	Exposure to ambient fine particulate pollution increased risk; association did not quite reach statistical significance and got weaker when controlling for nitrogen containing air pollutants	Over 10-year follow-up incidence rate ratio for hypertension for a 10 *μ*g/m^3^ increase in PM_2.5_ was 1.48 (95% CI, 0.95–2.31)

Johnson, 2009 [55] (cross-sectional)	132,224 National Health Interview Survey (NHIS)	USA	PM_2.5_ data from the US Environmental Protection Agency	Self-reported physician diagnosis of hypertension or use of medications	Odds ratio for prevalent hypertension was higher with higher levels of PM_2.5_ in Whites and not Blacks	Amongst Whites, a 10 *μ*g/m^3^ increase in PM_2.5 _exposure was associated with a small elevated risk of hypertension (adjusted odds ratio (OR) 1.05, 95% confidence interval (CI) 1.04–1.17); OR in Blacks was 0.90 (95% CI: 0.79–1.03)

Sørensen, 2012 [64] (cross-sectional and prospective)	57,053	Denmark	Dispersion model to calculate residential long-term nitrogen oxide	Self-reported incident hypertension was assessed by questionnaire	Nitrogen oxide (a measure of traffic air pollution that correlates well with fine particles and is easier to measure) was inversely associated with systolic and diastolic BP and the prevalence of self-reported hypertension, and there was no association with the risk of incident self-reported hypertension during approximately 5 years of follow-up	There were 0.53 mmHg and 0.50 mmHg decrease in systolic BP with nitrogen oxide exposure during 1- and 5-year periods preceding enrollment, respectively; the OR of self-reported hypertension with long-term exposure was 0.96 (95% CI: 0.91, 1.00)

Chuang, 2010 [57] (cross-sectional)	26,685	Taiwan	Monitoring stations by Taiwan Environmental Protection Agency	Measured BP	PM_10_ was associated with elevated systolic BP, triglyceride, apolipoprotein B, hemoglobin A1c, and reduced high-density lipoprotein cholesterol; elevated ozone was associated with increased diastolic BP	Increase of 0.47 mmHg; (95% CI, −0.09 to 1.02) with each interquartile range (34 *μ*g/m^3^) PM_10_

Schwartz, 2012 [63] (longitudinal)	853 elderly VA patients	USA	Traffic black soot	Measured BP	Increase in black soot was associated with increase in systolic and diastolic BP	An IQR increase in 1-year average black soot exposure (0.32 *μ*g/m^3^) was associated with a 2.64 mmHg increase in systolic blood pressure (95% CI 1.47 to 3.80) and a 2.41 mmHg increase in diastolic blood pressure (95% CI 1.77 to 3.05)

**Table 1c:** (c) Environmental noise and sleep quality

First author, year (design)	Sample size	Country	Noise assessment method	Sleep quality assessment method	Main finding	Effect size: ES *r* (95% CI)
Saremi, 2008 [67] (cross-sectional)	38	France	Recorded train noise at 40–50 dB(A)	Polysomnography	Arousal responsiveness increased with sound levels; awakenings (>10 s) were produced more frequently by freight train (compared to automotive/passenger)	Increase in noise level had main effect on the percentage of awakenings (*F*(2,50) = 26.94, *P* < 0.00001), and microarousals (*F*(2,50) = 64.29, *P* < 0.00001); there were increase in sleep fragmentation (*P* < 0.001) and a shorter arousal onset latency (*F*(1,36) = 16.18, *P* = 0.0002), with increasing noise level

Basner, 2011 [68] (cross-sectional)	72	Germany	Traffic noise events were recorded with class 1 sound level meters in bedrooms of residents living close to a road, a railway track, or an airport	Polysomnography, actigraphs, self-report	Subjective sleep assessment and recuperation were affected; indicators for sleep continuity were pronounced significantly except for awakening frequency	There were difficulty falling asleep (+89, *P* = 0.013), increased sleep disturbance (+126, *P* < 0.001), lighter sleep (+121, *P* < 0.001), and less recuperative sleep (+111, *P* < 0.001) in triple compared to single night exposure to noise; slow-wave sleep latency was 5.2 min longer in triple than single exposure night; REM latency was 9.0 min longer with the same exposure; time spent in REM was shorter in the triple than single or double exposure nights

Basner, 2005 [72] (cross-sectional)	128	Germany	Noise (45–80 dB(A)) was recorded with class 1 sound level meters (NC-10, Cortex Industries) in the vicinity of Dusseldorf Airport with closed or tilted windows	Polysomnography	Aircraft noise was associated with signs of sleep fragmentation (increased stage 1 and number of awakenings)	Slow-wave sleep was significantly reduced by 5.3 min, and total sleep time increased on average by 2.5 min

Agarwal, 2011 [71] (cross-sectional)	550	India	Self-report and objective assessment of noise indices (traffic volume and associated noise)	Self-report	Reported loss of sleep as a result of noise pollution	67% of respondents reported loss of sleep

Griefahn, 2006 [73] (experimental)	32	Germany	Noise range 32–74 dB(A) was applied	Polysomnography, self-report	Subjectively evaluated sleep quality decreased gradually with increasing noise levels; SWS latency prolongation, total sleep time reduction, and decrease of SWS during first sleep cycle were significant	The SWS latency and waketime after sleep onset were increased; total sleep time (TST) and sleep efficiency were decreased; In relation to sleep period time (SPT), the amount of time awake and in stage S1 (S0 and 1) was increased (+13 min), but REM-sleep and SWS were decreased (−11.7 min)

Horne, 1994 [82] (cross-sectional)	400	UK Airports	Aircraft noise event (ANE) was unit of measure	Wrist actigraphs and sleep logs	Minority of ANEs disrupted sleep; domestic idiosyncratic factors had greater impact on sleep	Effect size not available for qualitative-type study

Öhrström, 2004 [80] (literature review)	—	Sweden	Nordic prediction method for road traffic	Sleep survey	Reduction in road traffic after improvement in road traffic pattern resulted in improved self-reported sleep quality	Noise reduction from range of 56–69 dB(A) to 44–57 dB(A) resulted in improvement in self-reported sleep quality

**Table 1d:** (d) Air pollution and sleep quality

First author, year (design)	Sample size	Country	Air pollution assessment	Sleep quality assessment	Main finding	Effect size: ES *r* (95% CI)
Zanobetti, 2010 [51] (cross-sectional)	6441	USA	PM_10_	Polysomnography	Air pollution associated with increases in respiratory disturbance index and decrease in sleep efficiency	In the summer period, for every interquartile increase in short-term PM_10_ levels, there were 12.9% increase (95% CI: 2.77, 24.09) in RDI, 19.4% increase (95% CI: 3.67, 37.5) in percentage of sleep time at <90% oxygen saturation, and 1.20% decrease (95% CI: −2.40, −0.004) in sleep efficiency

Fang, 2014 [76] (cross-sectional)	3,821	USA	Black carbon (BC)	Self-report, Berlin Sleep Questionnaire	Increased sleep duration with annual BC in Blacks with no observation in Whites and Hispanics; sleep duration decreased in men and those with low socioeconomic status (SES) per IQR increase in BC but not in women and those with medium or high SES	OR for short sleep in men is 1.7 per IQR increase in BC (95% CI: 1.1, 2.6) and 1.6 (95% CI: 1.1, 2.3) for low socioeconomic status; OR for short sleep in Hispanics is 1.4 (95% CI: 1.1, 1.8); Blacks experienced increased sleep duration with increasing BC (*β* = 0.34 per IQR; 95% CI: 0.12, 0.57)

Abou-Khadra, 2013 [75] (cross-sectional)	276	Egypt	PM_10_	Self-report	PM_10_ and disorder of initiation and maintaining sleep were significantly associated (*P* = 0.012) and sleep hyperhidrosis was 0.045; PM_10_ and global sleep disturbance were marginally associated (*P* = 0.073)	Effect size was not reported
